# New potential instrument to fight hepatocellular cancer by restoring p53

**Published:** 2011-05-01

**Authors:** Franklin C. Vincent, Marek J. Los

**Affiliations:** 1 Interfaculty Institute of Biochemistry (IFIB), Tubingen University, Tubingen, Germany; 2 BioApplications Enterprises, Winnipeg, Manitoba, Canada; 3 Department of Clinical and Experimental Medicine (IKE), Integrative Regenerative Medicine Center (IGEN), Linkoping University, Linkoping, Sweden

**Keywords:** Annonaceous acetogenins, HBV X protein, Hepatocellular carcinomas, Toll like receptors

Although the name for cancer as a disease was coined by ancient Greeks and Persians, and in spite of billions of euros, dollars, and other currencies invested in cancer research, the disease remains a major killer, particularly in Western countries [1]. Over half of anticancer drugs, including acetogenins, alkaloids, and terpenes, are derived from natural products, especially from the plant kingdom [2][3][4][5][6]. Annonaceous acetogenins (ACGs) include a series of natural products isolated from Annonaceae plants. ACGs are white, waxy derivatives of long-chain (C35 or C37) fatty acids, characterized by a long aliphatic chain bearing a terminal methyl substituted α, β -unsaturated γ-lactone ring with one-, two- or three tetrahydropyran (THP) rings [5]. ACGs are a likely source of potential drugs as they exhibit various biological activities such as cytotoxic, antitumor, antiparasitic, pesticidal, pisicidal, antihelmenthic, antiviral, antimicrobial, and immunosuppressive activities. They are known to be powerful inhibitors of complex I (NADH: biquinone oxidoreductases) in the mitochondrial electron transport system. ACGs are also potential inhibitors of NADH oxidase in the plasma membranes of cancer cells and induce apoptosis by depleting ATP levels and arresting the cell cycle at the G1 phase [7]. Desacetyluvaricin is an acetogenin isolated from Rollina mucosa, a tree found widely in tropical America [8]. The fruit of this plant, commonly known in Mexico as "anonillo," "anonita del monte," and "cherimoya," is edible, and is utilized in folk medicine as a therapeutic agent [9]. Hepatocellular carcinoma (HCC), which accounts for 90% of primary liver cancer, is the third largest cause of cancer deaths worldwide, particularly in Africa and Eastern Asia. Mechanisms involved in the development of HCC are difficult to elucidate. Among various factors involved, research has reported that a particular Hepatitis B viral X protein (HBx) or Hepatitis C viral core proteins induce HCC without other oncogenic alterations in murine models [10] by activating NF-κB and AP-1 [11].

Hepatocellular carcinoma may progress through inactivation (direct interaction, mutation, and transcriptional repression) of the p53 tumor suppressor. HBx represses the transcription of the human p53 gene through the E-box element [12][13]. As the p53 protein binds and represses the HBV enhancer/X promoter, HBx repression of the p53-promotor triggers a positive response that further represses p53 expression [14]. Beside reciprocal transcriptional repression, HBx and p53 can inhibit each other by direct protein-protein interaction. The balance of the reciprocal inhibition between these two proteins may play a decisive role in the development of HBV-related malignancies. In their paper published in this issue, He and colleagues have tested the antitumor activity of desacetyluvaricin using the Hepg2.2.15 cell line. Flow cytometry analysis revealed a higher expression of p53 in desacetyluvaricin-treated cell lines when compared to untreated cell lines [15]. The increase of p53 in the presence of desacetyluvaricin is very promising and may open new avenues for the therapeutic intervention of hepatocarcinoma.

Another interesting aspect of desacetyluvaricin's activity that He and colleagues discovered is its effect on Toll-like receptors (TLRs). TLRs may promote tumor progression by acting directly on cancer cells, resulting in increased tumor cell-endothelial cell adhesion, tumour cell-extracellular matrix adhesion, and tumor cell-extracellular matrix invasion through NF-κB-mediated upregulation of β-1 integrin. Additionally, reports have demonstrated that TLR signaling pathways play a key role in activating stem-cell/progenitor proliferation and conversion to cancer-stem-cell-based liver tumor formation [16]. TLRs have also been found on tumor cells, but their role in these cells is still unclear. In some tumor types, TLRs promote tumor proliferation and survival, whereas in others TLR2, -3, and -9 are directly involved in apoptosis [17]. In their paper, He et al. reported that the expression of TLR4 is upregulated in the presence of the drug desacetyluvaricin [15]. Although they assume that in this context TLR4 helps to activate the innate and adaptive immune responses to tumors, one should not disregard the fact that TLR activation may be a double-edged sword with both antitumor and protumor consequences. It is therefore necessary to conduct comprehensive studies to assess the significance of TLR4 expression in tumor immunotherapy. Another aspect omitted from the He et al. study, and it would certainly draw additional interest to their work, is the potential effect of desacetyluvaricin on hepatocellular-cancer stem cells [18][19]. In conclusion, although the manuscript is rather preliminary, the observations made by He et al. underlines the importance of desacetyluvaricin and related compounds as potential leads for the development of new anticancer drugs.
